# Current Update on Intrinsic and Acquired Colistin Resistance Mechanisms in Bacteria

**DOI:** 10.3389/fmed.2021.677720

**Published:** 2021-08-12

**Authors:** Firdoos Ahmad Gogry, Mohammad Tahir Siddiqui, Insha Sultan, Qazi Mohd. Rizwanul Haq

**Affiliations:** Department of Biosciences, Jamia Millia Islamia, New Delhi, India

**Keywords:** chromosomal genes, *mcr*, electrostatic interaction, lipopolysaccharide, colistin resistance, *Enterobacteriaceae*

## Abstract

Colistin regained global interest as a consequence of the rising prevalence of multidrug-resistant Gram-negative *Enterobacteriaceae*. In parallel, colistin-resistant bacteria emerged in response to the unregulated use of this antibiotic. However, some Gram-negative species are intrinsically resistant to colistin activity, such as *Neisseria meningitides, Burkholderia* species, and *Proteus mirabilis*. Most identified colistin resistance usually involves modulation of lipid A that decreases or removes early charge-based interaction with colistin through up-regulation of multistep capsular polysaccharide expression. The membrane modifications occur by the addition of cationic phosphoethanolamine (pEtN) or 4-amino-l-arabinose on lipid A that results in decrease in the negative charge on the bacterial surface. Therefore, electrostatic interaction between polycationic colistin and lipopolysaccharide (LPS) is halted. It has been reported that these modifications on the bacterial surface occur due to overexpression of chromosomally mediated two-component system genes (PmrAB and PhoPQ) and mutation in lipid A biosynthesis genes that result in loss of the ability to produce lipid A and consequently LPS chain, thereafter recently identified variants of plasmid-borne genes (*mcr*-1 to *mcr-*10). It was hypothesized that *mcr* genes derived from intrinsically resistant environmental bacteria that carried chromosomal pmrC gene, a part of the pmrCAB operon, code three proteins *viz*. pEtN response regulator PmrA, sensor kinase protein PmrAB, and phosphotransferase PmrC. These plasmid-borne *mcr* genes become a serious concern as they assist in the dissemination of colistin resistance to other pathogenic bacteria. This review presents the progress of multiple strategies of colistin resistance mechanisms in bacteria, mainly focusing on surface changes of the outer membrane LPS structure and other resistance genetic determinants. New handier and versatile methods have been discussed for rapid detection of colistin resistance determinants and the latest approaches to revert colistin resistance that include the use of new drugs, drug combinations and inhibitors. Indeed, more investigations are required to identify the exact role of different colistin resistance determinants that will aid in developing new less toxic and potent drugs to treat bacterial infections. Therefore, colistin resistance should be considered a severe medical issue requiring multisectoral research with proper surveillance and suitable monitoring systems to report the dissemination rate of these resistant genes.

## Overview of Colistin and Emergence of Resistance

**Figure 1:**
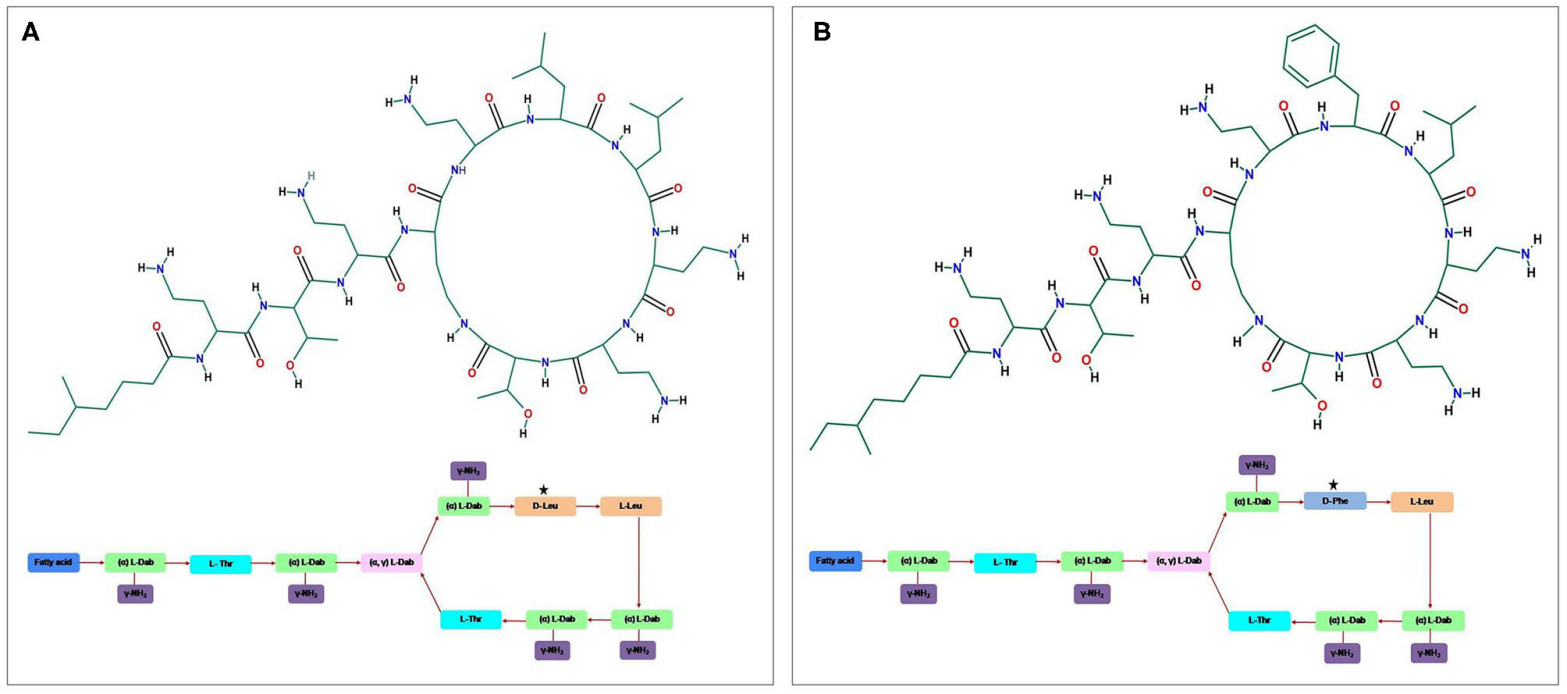
The general structure of the cyclic cationic peptide **(A)** polymyxin E (colistin) and **(B)** polymyxin B, phenylalanine (d-Phe) in polymyxin B peptide structure replaced with a leucine in colistin.

The antibiotics have been widely used in human, animal husbandry, and aquaculture, aiming to fight bacterial infections. The unmonitored and continued use of antibiotics has led to contamination of diversified environments, results in selective pressure on bacteria, and subsequently increases in the prevalence of antibiotic resistance (1–3). A steady increase in antibiotic resistance coupled with the decline in the development of new drugs is leading the world toward the pre–antibiotic era (4). This global public health threat requires immediate multidisciplinary steps to achieve the sustainable development goals, which are a collection of 17 interlinked global goals designed to be a roadmap for achieving a better and more sustainable future for all. Among these, Goal 3, i.e., Good Health and Well-being, was set up by the United Nations General Assembly to ensure healthy lives and promote well-being for all ages (5). New antibiotics active against Gram-positive bacteria provided some extent of respite (6, 7), but infections caused by antibiotic-resistant Gram-negative bacteria are emerging as a greater threat. Antibiotic-resistant bacteria and associated antibiotic resistance genes are gradually considered as diverse environmental contaminants. These antibiotic resistance genes are no longer limited to point sources, e.g., hospitals, sewage, and farms, but can also get disseminated in other relatively pristine environments, including rivers, lakes, and soils (8, 9). The occurrence of extremely drug-resistant and multidrug-resistant (MDR) bacteria has led to the reuse of polymyxin, a last-resort drug against severe bacterial infections (10–12). Polymyxins, non-ribosomal, cyclic oligopeptides antimicrobials structurally comprised a cyclic heptapeptide with five major chemical compounds: polymyxins A, B, C, D, and E. These compounds are differentiated based on variation in their amino acid sequences and fatty acid side chains. The prime representatives of polymyxin that have been used in clinical practice are polymyxin B and polymyxin E (colistin) (Figure 1) (12–16). Colistin is a polypeptide antibiotic isolated in 1947 from the bacterium *Paenibacillus polymyxa* subspecies *Colistinus* (17). Thereafter, it was reported from Japan (1947) that colistin is a secondary metabolite of the Gram-positive bacteria *P. polymyxa* subspecies *Colistinus* (18). In the 1950s, colistin was used as an intravenous formulation. In 1959, the US Food and Drug Administration approved colistin to treat various types of diarrhea and urinary tract infections. Failure of carbapenems against Gram-negative bacteria has led to the unprecedented increase in the use of colistin (one of the last-resort drugs) and subsequent emergence and dissemination of colistin resistance (14). Resistance to polymyxins has mainly emerged against polymyxin E class (colistin), a cationic polypeptide drug with cyclic decapeptide ring attached by an amide linkage to a fatty acyl chain, which is differentiated by single-amino-acid phenylalanine (d-Phe) in polymyxin B peptide structure with a leucine (d-Leu) in colistin (Figure 1) (16, 19). Until 2015, colistin resistance was known to be caused by chromosomal genes (phoPQ, pmrAB, and mgrB) (19–21). After the first report of plasmid-mediated *mcr*-1 gene from China in 2015 (14), more than 27 bacterial species have been identified from six continents (Asia, Europe, Africa, North America, South America, and Oceania). It is interesting to note that after 2015 the new reports about plasmid-mediated *mcr*-1 were made in isolates dating back to as early as 1980. The rise in the number of reports may be attributed to the long-term use of colistin in veterinary medicine. Similarly, global trade and travel to countries such Canada, United States, Japan, and Tunisia and overprescription of colistin in human medicine to treat highly resistant bacterial infection are likely reasons for colistin resistance (22–27). There is a high prevalence of colistin resistance that requires further studies to evaluate the factors involved, mechanism of acquisition, and dissemination (28, 29). We present herein an overview of recognition of alternative mechanisms of colistin action, the spread of acquired colistin resistance determinants, and diverse strategies taken by bacteria to extend resistance against colistin antibiotic.

## Mechanism of Colistin Activity

**Figure 2:**
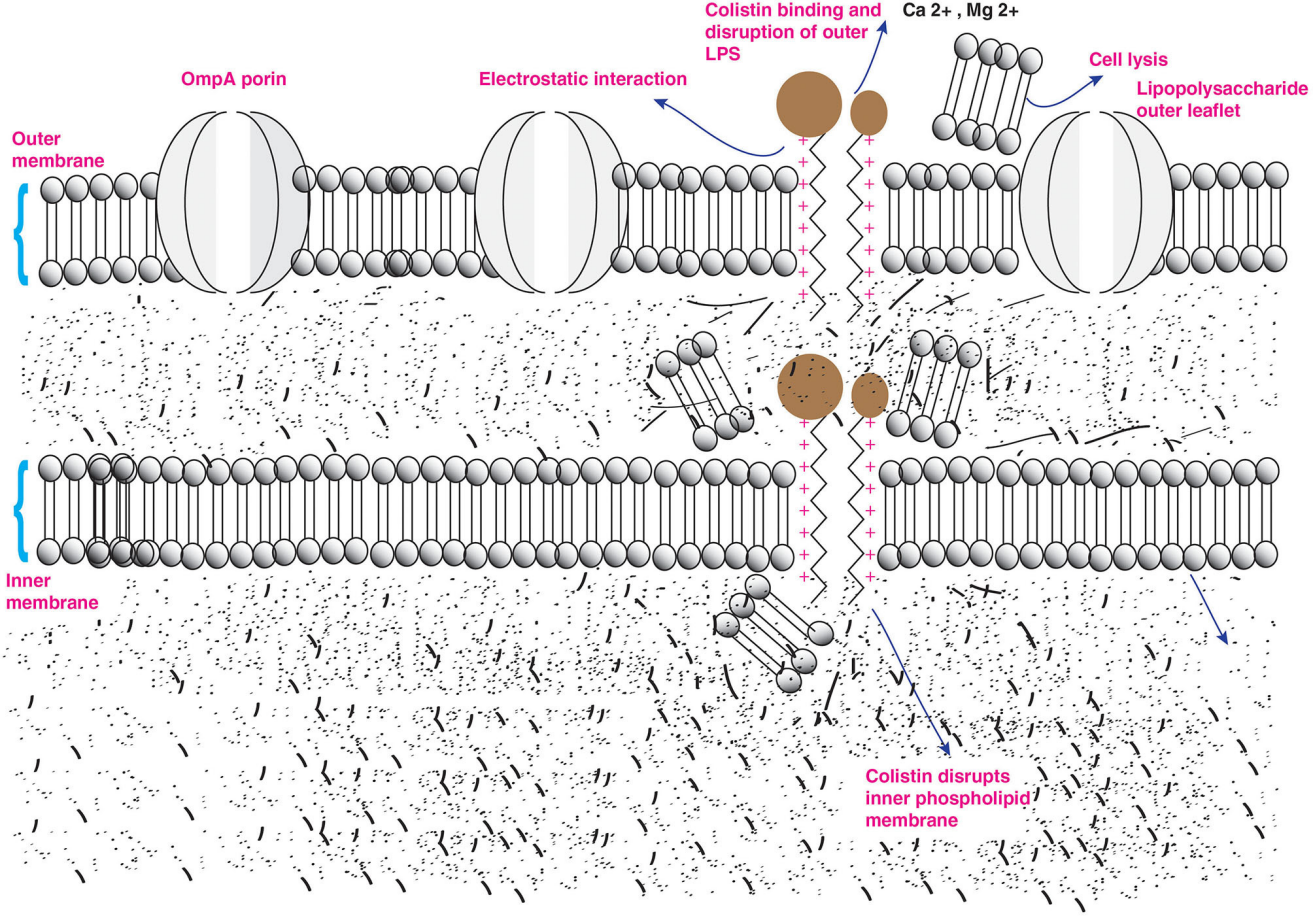
Action of colistin on bacterial membrane. The cationic cyclic decapeptide structure of colistin binds with the anionic LPS molecules by displacing calcium and magnesium from the outer cell membrane of Gram-negative bacteria, leading to permeability changes in the cell envelope and leakage of cell contents.

Antibacterial activity of colistin occurs on the outer membrane (OM) of Gram-negative bacteria. In addition, Gram-negative bacteria are well-characterized by the existence of an outer lipopolysaccharide (LPS) membrane that constitutes cell surface, which limits the entry of hydrophobic components and antibiotics (30, 31). Moreover, bacterial anionic LPS confers stability and integrity of the outer LPS membrane. But polymyxins are polycationic peptides crucial for their interaction with lipid A, a hydrophobic constituent of the LPS layer (32, 33). The antibacterial activity of colistin occurs through two-step mechanisms that are initial binding and employed permeabilization of the outer LPS membrane induces the displacement of Ca^2+^ and Mg^2+^ ions from the phosphate groups of LPS in a competitive way resulting in destabilizing cytoplasmic membrane, leading to disruption of the outer LPS and the loss of inner cellular contents, hence bacterial killing. The critical step of colistin action is based on the electrostatic interaction of cationic colistin peptide and anionic lipid A membrane also known as endotoxin component of LPS layer (Figure 2) (16, 19, 34, 35). Furthermore, It has been reported that bactericidal activity is independent of the passage of colistin into a bacterial cell (36) but inhibited in the presence of these divalent cations (33). However, LPS is the initial target for bacterial killing, but still, the exact mode of colistin action remains uncertain. Another antibacterial mechanism of colistin occurs by a potent antiendotoxin activity where the lipid A portion of LPS represents an endotoxin in Gram-negative bacteria. Therefore, colistin inhibits the endotoxin activity of lipid A by binding to and neutralizing the LPS molecules. This antibacterial activity mechanism occurs *in vivo* only (12, 37, 38). Moreover, another mechanism of action occurs by vital respiratory enzymes (type II NADH-quinone oxidoreductases NADH-2) inhibition by colistin drug in Gram-negative bacteria (39). The alternative strategy of colistin action occurs by induction of rapid cell death via hydroxyl radical production through colistin binding to the lipid membrane. The free radicals are generated when colistin crosses the OM and IM of LPS. The hydroxyl radical generation occurs via the production of the reactive oxygen species; hydroxyl radicals. (^•^OH), superoxide (${\text{O}}_{2}^{-}$), and hydrogen peroxide (H_2_O_2_), which cause oxidative stress. ${\text{O}}_{2}^{-}$ is generated when colistin enters into and crosses the OM and IM, followed by the conversion of ${\text{O}}_{2}^{-}$ into H_2_O_2_ by superoxide dismutase. After that, H_2_O_2_ oxidizes ferrous iron (Fe^2+^) into ferric iron (Fe^3+^), besides the formation of ^•^OH; this process is known as Fenton reaction. This reaction can induce oxidative damage in bacterial DNA, proteins, and lipids, leading to cell death. This mechanism of killing has been shown to occur in the colistin-sensitive and MDR isolates of *Acinetobacter baumannii* and *Escherichia coli* but does not take place in polymyxin-resistant strains (40, 41).

## Spectrum of Activity

Colistin has a narrow bactericidal activity spectrum against the most common Gram-negative *Enterobacteriaceae*. Colistin has antibacterial activity against members of the *Enterobacteriaceae* family, including *Klebsiella* species, *E. coli, Citrobacter* species, *Shigella* species, *Enterobacter* species, and *Salmonella* species. Colistin also has significant bactericidal activity against prevalent non-fermentative Gram-negative bacteria, including *Stenotrophomonas maltophilia, A. baumannii*, and *Pseudomonas aeruginosa* (12, 42, 43). Moreover, some species are resistant naturally to colistin, such as *Proteus* species, *Providencia* species, *Morganella morganii, Pseudomonas mallei, Chromobacterium* species, *Burkholderia cepacia, Serratia marcescens, Edwardsiella* species, *Campylobacter, Vibrio cholera*, and *Brucella, Legionella*. Moreover, colistin antibiotic is not active against Gram-negative cocci (*Neisseria* species), anaerobic bacteria (12, 35), and *Aeromonas* species (except *Aeromonas jandaei*), whereas *Aeromonas hydrophila* was found to have inducible resistance (44, 45).

## Pharmachemistry of Colistin

The pharmacokinetics (PK) and pharmacodynamics (PD) determine the related dosage and therapeutics of colistin. It is imperative to determine the effective dosage of a drug to treat dreadful infections. With increasing resistance, it has become necessary to study the PKPD relation of colistin to determine proper treatment regimen. Until now, PK and PD data of colistin are not well-reported, especially in patients suffering from renal replacement therapy (46, 47). Therefore, time-bound studies are needed further to understand the PD/PK relationship of colistin to determine fixed doses to critically ill patients. Randomized controlled trials are urgently required to clarify further the issues surrounding the efficacy and safety of colistin. Two decades ago, non-specific microbiological assays were applied to estimate the colistin concentrations in biological fluids (38). Moreover, it has been reported that bactericidal activity is dependent on colistin concentration (11, 48–50). The plasma colistin concentration of 2 μg/mL has been found to be a reasonable dose for bacteria having minimum inhibitory concentrations (MICs) of ≤1 μg/mL (51). Postantibiotic effect was noticed among *P. aeruginosa, A. baumannii*, and *Klebsiella pneumoniae*, although the main antibacterial activity is noticed only when susceptible strains are exposed to colistin. However, in *A. baumannii* and *K. pneumoniae*, regrowth has been reported for static time-kill studies (49, 50). It has also been reported that the emergence of colistin-resistant subpopulation is dependent on colistin heteroresistance that enables growth at ≥4 μg/mL of colistin within a sensitive population with a MIC of ≤2 μg/mL (52, 53), e.g., *K. pneumonia* (50, 54) *P. aeruginosa*, and *A. baumannii* (55).

## One Health Perspective for Colistin Resistance

Antimicrobial resistance is a public health problem of complex epidemiology suitable for a comprehensive study as part of the One Health perspective. One Health is defined as the collaborative effort of multiple disciplines working locally, nationally, and globally to attain optimal health for people, animals, and the environment through policy, research, education, and practice (56, 57). The drivers of antimicrobial resistance include antimicrobial use and abuse in humans, animals, and the environment followed by the dissemination of resistant bacteria and resistance determinants between and among these sectors around the globe. Major concerns in the animal health and agriculture sectors are mass medication of animals with antimicrobials that are critically important for humans, like colistin, one of the last-resort drugs to tackle Gram-negative bacterial infection. The unmonitored use of colistin in human and veterinary medicine results in the emergence of colistin resistance among *Enterobacteriaceae*. The emergence of *mcr*-1 has almost certainly been exacerbated by the use of colistin on Chinese and Southeast Asian farms (14) and subsequent spread to dozens of other countries (35, 58, 59). The colistin resistance has risen in Spain, Italy, and Greece with 31, 43, and 20.8%, respectively (60–62). Colistin resistance makes it difficult to use it as a therapeutic option for multidrug-resistant bacteria. Therefore, the use of colistin is dependent on the type of infection, sensitivity phenotype of bacteria, and the target of PK/PD antibiotic, including possible side effects (63). The most adverse effects of colistin are nephrotoxicity and neurotoxicity due to parenteral use (18, 64, 65). In addition to human medicine, colistin has been widely used to prevent and treat various veterinary infectious diseases. Colistin use in veterinary varies extensively among different countries, e.g., in Spain, it is used during lactation, gestation, and control of metaphylactic intestinal diseases (66). In France, Austria, and Sweden, colistin is used during the postweaning time in pig farms (67–69). A German and Netherlands study reported that colistin is the most widely used antibiotic in animal farming next to trimethoprim/sulfonamides, tetracyclines, lincosamides, and macrolides (70). In Asian countries, colistin and other antibiotics are used extensively for veterinary infections and agriculture purposes (71, 72). The increasing trend of colistin consumption is assumed to reach 16,000 tons in China by the end of 2021 (72). There are reports of colistin resistance in the *Enterobacteriaceae* from fruits and vegetable samples (73–77). Reports revealed the association between the incidence of food-borne diseases and the food production chain that occurs via eating contaminated raw vegetables (9, 73–75, 78).

The emergence and rapid geographic dissemination of colistin-resistant bacteria and colistin resistance genes have become a health concern. Therefore, an integrated and holistic multisectoral approach is the need of the hour to combat colistin resistance, in particular, better integration of human health, veterinary, and environment (79–81). Several countries and international agencies have included a One Health perspective within their action plans to address antimicrobial resistance. Necessary actions include improvements in antimicrobial use regulation and policy, surveillance, stewardship, infection control, sanitation, animal husbandry, and alternatives to antimicrobials. World Health Organization (WHO) has recently launched new guidelines on the use of medically important antimicrobials in food-producing animals, recommending that farmers and the food industry should stop using antimicrobials routinely to promote growth and to prevent disease in healthy animals. These guidelines aim to preserve the effectiveness of antimicrobials that are important for human medicine by reducing their use in animals (82–84). Different monitoring systems were established in European countries for surveillance to check colistin consumption and the emergence of resistance (85–87). Recent multinational strategies to address the urgency of AMR include the US National Action Plan for combating antibiotic-resistant bacteria and WHO Global Action Plan on Antimicrobial Resistance (88, 89). WHO's GLASS (Global Antimicrobial Resistance Surveillance System) is helping countries strengthen national surveillance systems and provides more comprehensive standardized AMR surveillance data (90). The countries and networks at the forefront of AMR efforts should engage additional stakeholders in developing an effective strategy that will have far-reaching benefits in minimizing the impact of this urgent problem on human and animal health, environment, global economy and national and global security.

## Challenges of Testing and Detecting Colistin Resistance Determinants

It is critical to design phenotypic tests capable of detecting colistin resistance in Gram-negative bacteria. Until now, there was no agreement on the methodology for colistin susceptibility testing. Because of the weak diffusion of colistin in agar, the disc diffusion process and gradient tests were inaccurate (91–95). As a result, disc diffusion and gradient diffusion are ineffective in determining polymyxin susceptibility. Both the European Committee on Antimicrobial Susceptibility Testing (EUCAST) and the Clinical and Laboratory Standards Institute recommended the International Standard Organization 20776 standard broth dilution method for testing colistin MIC values (96, 97). The reference broth microdilution method, on the other hand, is difficult to apply in routine microbiological diagnostics. The EUCAST does not recommend using automated systems to determine the phenotype of bacterial sensitivity, such as Vitek 2 (bioMérieux, France), WalkAway (Beckman Coulter, USA) or BD Phoenix (Becton Dickinson, USA) for the analysis of Gram-negative bacteria sensitivity to colistin. This is because these systems' accuracy in determining colistin MIC is minimal compared to the reference method especially in the 2–4-mg/L range (98–101). There is currently insufficient understanding of acquired colistin resistance mechanisms to design a sensitive molecular test specific enough to be recommended as best practice. Genotypic methods, in particular, are unlikely to detect any of the chromosomal defects known to cause most phenotypic colistin resistance in clinical settings (20). A negative polymerase chain reaction (PCR) molecular test result cannot be used to predict colistin susceptibility because the test cannot rule out the existence of chromosomal mechanisms of resistance or even novel *mcr* genes not included in the test. As evidence of this limitation, high colistin resistance rates have been reported among *K. pneumoniae* strains that produce carbapenemase but lack *mcr* genes (102–105). In these circumstances, a negative PCR result for mcr genes would have poor predictive value for a colistin-susceptible phenotype. However, if the results are intended to guide clinical management, inference of phenotype based solely on a genotypic result may be valid only when the genotypic result is positive (i.e., mechanisms or genes detected) with the caveat that the resistance may not be detected.

## Methods for Rapid Detection of Colistin Resistance

The rapid polymyxin NP is an innovative technique for identification of colistin resistance among Gram-negative bacteria (98). The researchers are currently working on tests to detect colistin resistance in non-fermenting bacilli. In the presence of a given concentration of polymyxin E and B, the rapid polymyxin NP test detects glucose fermentation associated with bacterial growth; the presence of acid metabolites is shown by a shift in pH and the color of the indicator (red phenol) turning from orange to yellow. The test's sensitivity and specificity are similar to the reference broth microdilution method (99.3 and 95.4%, respectively). This test is simple to perform and yields a result in <2 h (98). Chromogenic media are widely used for screening because they enable bacteria to develop as properly colored colonies. The super polymyxin screening medium was the first agar medium for detecting colistin-resistant Gram-negative rods from bacterial cultures and rectal swab samples (106). The commercial version of this medium is super polymyxin medium (ELITechGroup, Puteaux, France) for the identification of colistin-resistant *Enterobacterales* strains, including those with the low MIC values (mg/L) that contain the *mcr-*1 gene (107). It consists of methylene blue agar and includes colistin, daptomycin, and amphotericin B at concentrations (3.5, 10, and 5 g/mL, respectively). The other medium, CHROMagar COL-APSE medium was used to identify colistin-resistant bacteria (108); this medium distinguishes colistin-resistant *Enterobacterales* strains from non-fermenting rods. The LBJMR medium is a new polyvalent culture medium for the isolation and selection of colistin-resistant bacteria and vancomycin-resistant bacteria (109). This medium was developed by combining colistin sulfate salt (4 g/mL), vancomycin (50 g/mL), and a fermentation substrate (7.5 g/L of glucose) with purple agar base (31 g/L). Moreover, new chromogenic medium, CHROMID Colistin R agar (COLR; bioMérieux, France), was introduced in the market in early 2018, allowing the screening of colistin-resistant *Enterobacteriaceae* in clinical samples such as rectal swabs and stools. The COLR is a manual qualitative diagnostic test that distinguishes colistin-resistant isolates from susceptible isolates. Colistin-resistant strains form colored colonies on chromogenic media, with the color varying according to the species. However, colistin-susceptible isolates, on the other hand, do not grow on the COLR plate (110). The chromogenic method is based on agar dilution. Still, EUCAST does not recommend it for determining bacterial susceptibility to colistin because the detectability threshold increases with the growth of the bacterial inoculums (100). Moreover, Turlej-Rogacka et al. (111) reported that when compared to broth dilution methods, the agar dilution method yields more accurate results in evaluating colistin MIC values (111). Behera et al. (93) confirmed the strong correlation between the reference and agar dilution methods (93, 94). The biggest challenge is the adhesion of colistin to plastic during handling (112). According to the above authors, the agar dilution process reduces the colistin plastic–binding process significantly, and the MIC results obtained by the agar dilution method are exact (93, 112, 113). The COLR medium uses the borderline concentrations of colistin to qualify strains as susceptible or resistant. This chromogenic medium is a qualitative detection method for *Enterobacteriaceae* and does not permit colistin MIC values to be determined against the test bacterial strains; thus, it should be considered as a screening test only. The clinical interpretation is, by contrast, significant in the treatment of infections caused by colistin-resistant bacteria. This means that the colistin resistance is categorized rather than the MIC value determined as maximum dosages are prescribed irrespective of the precise sensitivity levels. However, MIC values are important to monitor the rise in colistin resistance in Gram-negative bacteria. Other new-generation methods for detecting colistin resistible strains were recently developed, e.g., loop-mediated isothermal amplification (LAMP) for nucleic acid detection (114) and CT103XL microarray (115). It has been demonstrated the sensitivity of the LAMP test is 10 times higher than conventional PCR and confirmed its usefulness in the detection of the *mcr*-1 gene from *Enterobacterales* (114). Similarly, the new microarray CT103xl has been demonstrated by simultaneously identifying *mcr*-1, *mcr*-2, and clinically important ESBL genes (115). Whole-genome sequencing would allow screening for *mcr* genes and known chromosomal mutations that confer colistin resistance. Bioinformatics analysis could be conducted by applying the Center for Genomic Epidemiology Web tools (116) and ResFinder 4.0 (117). Although the sensitivity and negative predictive value would be affected by the inclusion of strains with novel mechanisms of resistance, this is the most comprehensive method for detecting all currently known putative colistin resistance mechanisms. It will also enable a retrospective analysis of sequencing data as new resistance mechanisms are described. Whatever the molecular method used, it is critically important to ensure that either the PCR detects all currently known *mcr* genes or the databases used to impute resistance mechanisms from whole-genome sequencing data are up to date. As our understanding of colistin resistance mechanisms improves, so will the concordance between phenotypic and genotypic test results. As for many other antimicrobial agents, molecular testing may eventually offer an alternative to phenotypic testing for the surveillance of colistin resistance.

## Resistance Mechanisms in *Enterobacteriaceae*

### Mechanisms of Intrinsic Resistance

Resistance to colistin occurs naturally in *S. marcescens* and *Proteus mirabilis* by arnBCADTEF and eptB gene expression and consequently addition of phosphoethanolamine (pEtN) and 4-amino-4-deoxy-l-arabinose (l-Ara4N) cationic groups on LPS, respectively. This modulation increases the cationic charge on the LPS membrane, which is the initial target of the colistin. It therefore decreases colistin antibiotic binding results in intrinsic resistance of these bacterial strains (12, 28, 118, 119).

### Acquired Resistance in *Enterobacteriaceae*

#### Chromosomal Modulation of PmrAB and PhoPQ Two-Component Systems

**Figure 3:**
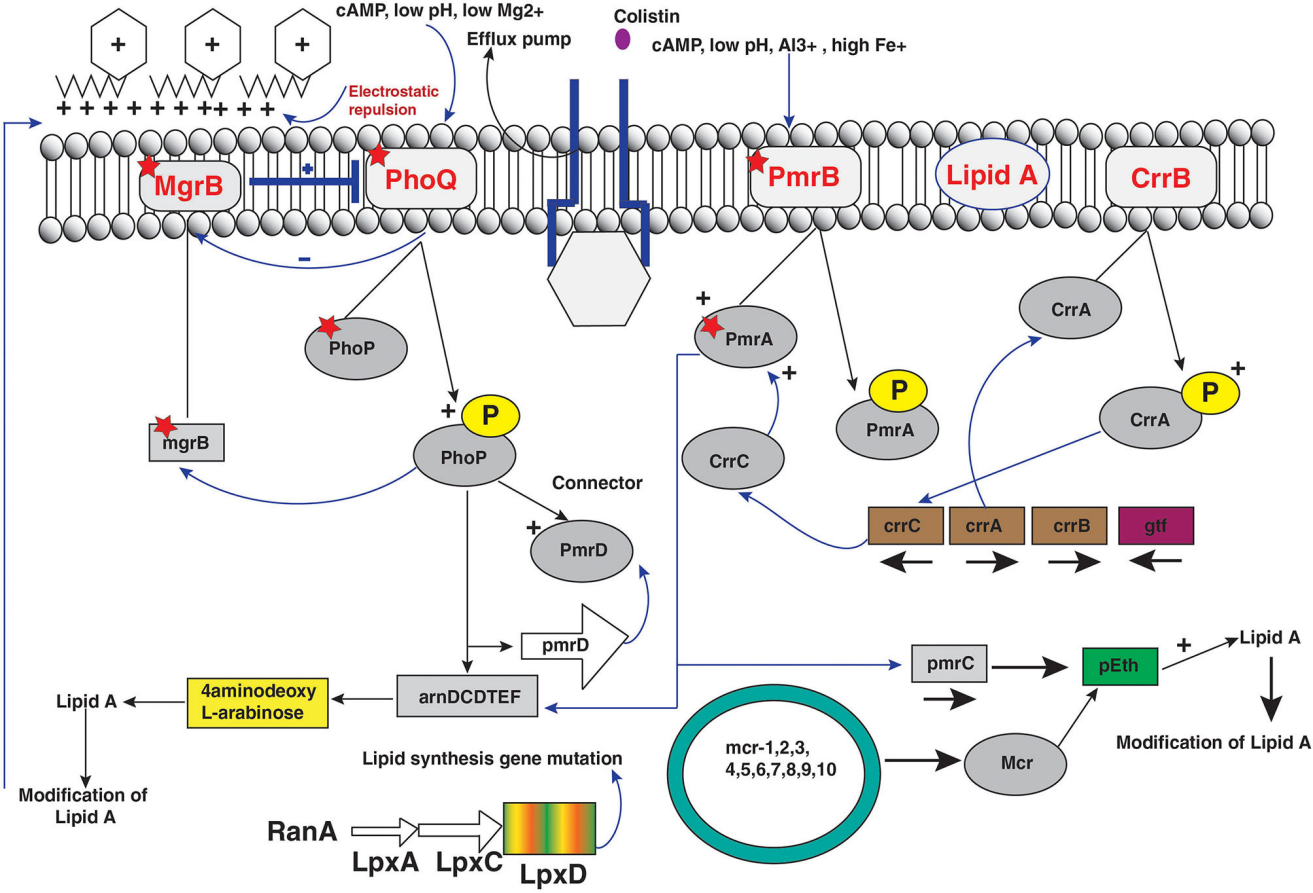
Regulators of colistin resistance mechanisms via chromosomal and plasmid-mediated pathways of lipopolysaccharide modifications in *Enterobacteriaceae*.

The acquired colistin resistance has been reported in some Gram-negative bacteria such as *Enterobacter, E. coli, Salmonella*, and *K. pneumoniae* but remains unclear for some other bacterial strains (35, 120). Colistin resistance mechanism occurs by chromosomal modulations similar to bacteria that are naturally resistant to colistin. The various molecular mechanisms have been determined, and the most common modifications occur via cationic groups (l-Ara4N and pEtN) to the lipid membrane of bacterial strains (14, 35). The several operons and genes are related to the LPS membrane modulations (Figure 3). The two-component systems PhoPQ and PmrAB are extensively responsible for LPS modifications by addition of cationic groups to the LPS membrane. The operons coding enzymes are responsible for modifications in pmrC gene, pmrE gene, and the pmrHFIJKLM operon regulatory genes. Moreover, two-component systems are regulated by the mgrB gene, which negatively controls the expression of the two-component PhoPQ system. The pmrCAB operon codes three proteins: (a) the pEtN response regulator PmrA, (b) sensor kinase protein PmrAB, and (c) phosphotransferase PmrC (121, 122). However, the synthesis of the l-aminoarabinose group on LPS occurs by activation of pmrHFIJKLM and pmrE gene expression (123). Likewise, PmrAB two-component regulatory system encoded by PmrA and pmrB is activated by various environmental stimuli such as low pH (5.5), ferric (Fe^3+^) iron, macrophage phagosomes aluminum(Al^3+^), etc., and results in PmrB activation via periplasmic domain (121). In turn, pmrB activates PmrA by phosphorylation through tyrosine kinase protein of the pmrB gene. The PmrA activates transcription of the pmrCAB operon and the attached pmrE gene result in LPS modifications with the addition of cationic pEtN and l-Ara4N moieties (Figure 3) (121, 124). Reportedly, Specific mutations within the PmrA and pmrB genes have been found responsible for acquired colistin antibiotic resistance in *K. pneumoniae* (13, 98, 125–127), *Enterobacter aerogenes* (128), and *Salmonella enterica* (129, 130) (Table 1). Another PhoPQ two-component system encoded by PhoP and PhoQ genes, which expresses two proteins: (a) regulator protein PhoP and (b) sensor protein kinase PhoQ. The PhoPQ system is activated in acidic conditions (low pH) by various environmental stimuli such as low magnesium (Mg^2+^) and macrophage phagosomes and intercede PhoQ activation through its periplasmic lipid domain (124). Moreover, transcription activation of the pmrHFIJKLM operon occurs by PhoP resulting in l-Ara4N addition to the LPS membrane (180, 181). The PmrA protein is also activated by the PhoP gene either directly or indirectly via the PmrD (connector protein), causing the addition of pEtN to the LPS. However, acquired colistin resistance was reported in *K. pneumoniae* by mutations in the PhoP and PhoQ genes (13, 98, 126, 140, 182, 183) (Table 1). It has been observed in *E. coli* that a potential mutation in PmrAB causes acquired colistin resistance (131). The sum of constitutive activation of PhoPQ through modulations causes overexpression of the pmrHFIJKLM operon, thus synthesizing lipid-modifying moieties l-Ara4N that binds to lipid A membrane.

**Table 1:** Characteristics of colistin resistance mechanisms and modifications associated with most known bacteria.

**Family**	**Bacteria**	**Genes/determinants**	**Resistance mechanisms**	**References**
*Enterobacteriaceae*		pmrA/pmrB	Modification of lipid A by arnBCADTEF operon, pmrC and pmrE genes	(98)
		phoP/phoQ	Modification of lipid A by activation of the pmrHFIJKLM operon/activation of pmrAB by pmrD	(131)
	*E. coli*	arnBCADTEF	Modification of lipid A by pEtN and l-4AraN	(35)
		mgrB mutation	Overexpression of phoPQ and activation of pmrHFIJKLM	(132)
		*acrB* mutation	Efflux pump	(133)
		*mcr*-1,2,3,4,5, and *mcr*-9	Phosphoethanolamine transferase	(21, 134–139)
		pmrA/pmrB	Modification of lipid A by arnBCADTEF operon, pmrC, and pmrE genes	(127)
		phoP/phoQ	Modification of lipid A by activation of the pmrHFIJKLM operon/activation of pmrAB by pmrD	(140)
		arnBCADTEF	Modification of lipid A by pEtN and l-4AraN	(35)
	*K. pneumoniae*	mgrB mutation	Overexpression of phoPQ and activation of pmrHFIJKLM	(21, 132)
		crrB mutation	Modification of lipid A by upregulation of pmrAB/activation of the glycosyltransferase	(141)
		ramA	Modulates lipid A biosynthesis	(37, 142)
		*acrB* mutation	Efflux pump	(133)
		*mcr*-1,7 and 8	Phosphoethanolamine transferase	(143–145)
		arnBCADTEF	Modifications of the LPS moiety l-Ara4N and/or PEtN modification of lipid A	(146, 147)
	*S. enterica*	pmrAB, phoPQ	Activation of the two-component system	(35)
		*mcr*-1,2,3,4,5, and *mcr*-9	Phosphoethanolamine transferase	(110, 148–152)
		pmrA/pmrB	Modification of lipid A by activation of the pmrHFIJKLM operon/activation of pmrAB by pmrD	(128)
	*Enterobacter* species	phoP/phoQ	Modification of lipid A by pEtN and l-4AraN	(153)
		*arnBCADTEF*	Overexpression of phoPQ and activation of pmrHFIJKLM	(153)
		*mcr*-1,4,5and 10	Phosphoethanolamine transferase	(29, 149, 154, 155)
	*Citrobacter freundii*	*mcr-*1 and 3	Phosphoethanolamine transferase	(156, 157)
*Aeromonadaceae*	*Aeromonas* species	*mcr-*1,3, and 5	Phosphoethanolamine transferase	(134, 158–160)
		pmrF operon	Modifications of the LPS moiety by l-Ara4N biosynthesis	(161)
		lpxA, lpxC, lpxD	Inactivation of lipid A biosynthesis abolishing LPS synthesis	(19, 162, 163)
		pmrAB	Modification of lipid A by arnBCADTEF operon, pmrC, and pmrE genes	(147, 164, 165)
*Moraxellaceae*	*A. baumannii*	adeABC, HlyD family, emrA, emrB	Efflux pump	(162, 166, 167)
		*mcr*-1,2,3, and 4	Phosphoethanolamine transferase	(168, 169)
		colR/colS, cprRS	LPS additions in response to high Zn^2+^ modifications of the LPS moiety	(35, 170)
*Pseudomonadaceae*	*P. aeruginosa*	pmrAB, phoPQ	LPS additions in response to low Zn^2+^	(35, 171)
		*mcr*-1 and 2	Phosphoethanolamine transferase	(172, 173)
		arnBCADTEF	Modification of lipid A by pEtN and l-4AraN	(174)
*Morganellaceae*	*P. mirabilis*	SapABCDF	Mutation Efflux pump	(175)
		*mcr*-3	Phosphoethanolamine transferase	(176)
*Yersiniaceae*	*S. marcescens*	arnBCADTEF	Modification of lipid A by pEtN and l-4AraN	(177)
*Helicobacteraceae*	*Helicobacter pylori*	Cgt	Alterations in membrane composition modification of lipid A	(35)
*Vibrionaceae*	*V. cholera*	gspIEF, lpxN	Modifications of the LPS Moiety	(178)
*Pasteurellaceae*	*Haemophilus influenzae*	lic1/2A, lpsA, lgtF, opsX	colistin resistance mechanism	(179)
*Burkholderiaceae*	*Burkholderia multivorans*	buml_2133/2134	Membrane fluidity/permeability	(178)

#### CrrAB Two-Component System

CrrAB is a two-component regulatory system modulating the PmrAB system. It encodes two protein products as CrrA as a regulatory protein and CrrB as a sensor kinase protein. Colistin resistance in *K. pneumoniae* was reported by mutation of the crrB part of two-component systems (183). The glycosyltransferase-like protein was expressed through mutation of the CrrB protein that leads to modification on the outer LPS membrane (183). However, colistin resistance was observed by six amino acid substitutions in two-component crrB protein with MIC range 512–2,048 μg/mL. The expression analysis of pmrHFIJKLM operon with pmrC and pmrE leads to overexpression of PmrAB operon indirectly controlled by mutation of crrB gene and hence excess production of cationic pEtN and 4-amino-4-deoxy-l-arabinose to lipid A membrane that leads to colistin resistance (141, 183). Additionally, CrrAB and PmrAB two-component systems are indirectly connected via CrrC; mutations in the crrB gene system result in increased expression of CrrC. The amino acid substitution on the crrB protein leads to higher activation of protein by autophosphorylation that results in colistin resistance (141).

#### MgrB Regulator of the PmrAB and PhoPQ Two-Component Systems

The regulator of PmrAB and PhoPQ two-component system gene mgrB encodes a 47-amino-acid transmembrane protein that exerts negative feedback regulation of the PhoPQ two-component system and inhibits the kinase activity of PhoQ resulting in repression of the PhoQ gene (132, 184, 185). However, upregulation of PhoPQ operon occurs by inactivation and mutation of mgrB gene and consequent activation of pmrHFIJKLM operon, leading to excess production of cationic l-Ara4N that results in blocking colistin binding to LPS membrane. The various missense and non-sense mutations resulted in amino acid substitutions with truncated mgrB protein causing acquired resistance to colistin in Gram-negative bacteria, particularly *K. pneumoniae*. Moreover, it has been reported that other modes of modifications such as deletion or insertion in the mgrB gene sequence cause complete elimination of the mgrB locus (182, 184). In addition, insertional inactivation of the mgrB gene was found by several insertion sequences belonging to various families and inserted at different positions in the mgrB gene locus (13, 76, 98, 182, 184–188). Recently, colistin resistance was reported by transposition of genes encoding carbapenemase and extended-spectrum of β-lactamases, which leads to chromosomal mgrB gene disruption (189, 190). Moreover, reports suggest that coselection of colistin resistance with β-lactamase genes occurs with selection pressure, and deletion of the mgrB gene led to upregulated expression of the PhoP gene in *E. coli* resulting in colistin resistance (132).

#### Resistance by a Mutation in LPS Synthesis Genes

Resistance to colistin antibiotic was reported by loss of the initial colistin and membrane binding by electrostatic interactions with lipid A component of LPS in Gram-negative bacteria (14, 35). The binding is lost by complete loss of LPS membrane target site for colistin antibiotic. The LPS synthesis is governed by ramA gene locus containing three subgenes: ramA, romA, and ramR. The ramA and romA genes were downregulated by ramR; moreover, ramA regulator is known to be present in some Gram-negative bacteria such as *Citrobacter* species, *Salmonella* species, *K. pneumoniae*, and *Enterobacter* species. However, ramA modifies lipid A membrane biosynthesis that regulates permeability barriers (191). Recently, it has been reported that higher levels of RamA cause LPS modulations and hence increased colistin resistance (191). The loss of LPS results in colistin resistance in *A. baumannii*. The mutations in lipid A biosynthesis genes, *lpxA, lpxC*, and *lpxD*, cause total loss of LPS production halting colistin binding to membrane and hence colistin resistance. There are mutations in the first three genes of the LPS production and therefore complete loss of the LPS layer (19, 192).

#### Role of the Capsule in Colistin Resistance

The capsule in bacteria acts as a defensive and protective covering against an antimicrobial peptide including colistin (20, 193), and capsular polysaccharide is released by the bacteria from their surface (194). However, it has been reported that resistance pattern is dependent on the number of capsule layers that the bacteria can produce. Gram-negative bacteria such as *K. pneumoniae* with multiple capsule layers were found more resistant than bacteria with only a few CPS layers (10, 195). Moreover, the unregulated expression of CPS syntheses decreases colistin electrostatic interaction with the target site in *K. pneumoniae*, resulting in increased colistin antibiotic resistance (193). Subsequently, there are Cpx (conjugative pilus expression) and Rcs (regulator of capsule synthesis) regulators of capsule layer formation located on the LPS membrane (196). It has been reported that two-component PhoPQ and efflux pump KpnEF are activated by Rcs and Cpx, respectively (20, 197). Additionally, the ugd gene was found in CPS and l-Ara4N biosynthesis via phosphorylation causing assembly of capsules in bacterial strains and hence colistin resistance (198, 199).

#### Role of Efflux Pumps

There are reports of efflux pumps such as KpnEF, AcrAB, and Sap proteins systems involved in colistin resistance among bacterial isolates. The activation of these efflux pumps resulted in the increase of colistin resistance (20, 189, 200–203). The KpnEF pump is a member of the Cpx regulon and belongs to the SMR protein family (10). It has been revealed that efflux pumps are activated by colistin resistance to other related antibiotics such as rifampicin, ceftriaxone, and erythromycin (201). Moreover, the mutation in KpnEF results in more susceptibility and double reduction in the MICs with colistin antibiotic (201). From the same pipeline, efflux pump AcrAB is a small part of the AcrAB-TolC multifaceted structure involved in colistin resistance. Accordingly, AcrAB-mutant *E. coli* revealed an 8-fold increase in colistin antibiotic sensitivity. In addition, it has been observed that PhoPQ TCS is dependent on the expression of AcrAB pump proteins (204). Similarly, SapABCDF operon constitutes five proteins in *P. mirabilis* resulted in increasing sensitivity to colistin by a mutation in Sap ABCDEF operon (175, 204). It has been shown that the use of efflux-pump inhibitors in the test medium carbonyl cyanide 3-chlorophenylhydrazone leads to a reduction in MIC for colistin-resistant strains (203).

#### Emergence of Plasmid-mediated *mcr*-Like Genes

**Figure 4:**
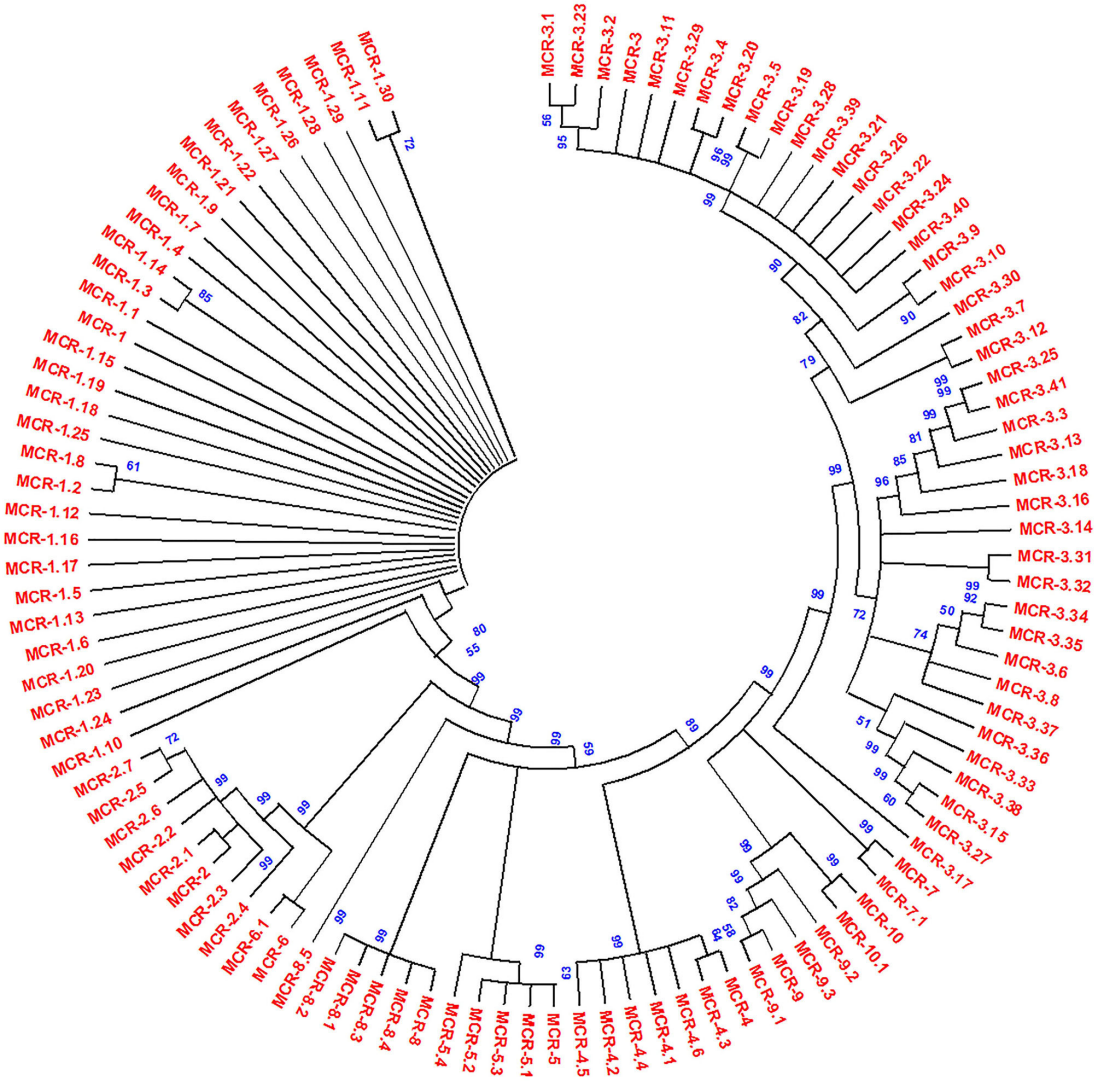
Phylogenetic tree of *mcr* gene variants, constructed by neighbor-joining method (bootstrap analysis with 500 replicates). All the proteins used for the phylogenetic tree were members of the PEA lipid A transferase family. Multiple sequence alignment was conducted using Clustal W, and resultant output was generated with Mega X.

The acquired resistance from chromosomal to plasmid DNA coded on transposable genetic elements on plasmids with *mcr*-1 and several variants has been reported first in *E. coli* from China; after that, plasmid-mediated *mcr*-1 and variants have been detected in other Gram-negative bacterial isolates (134, 148). The resistance pattern was found to be the same as the chromosomal pmrC gene that codes *mcr*-1 protein pEtN transferase. It was hypothesized that *mcr* genes derived from intrinsically resistant environmental bacteria, e.g., *Paenibacillus* species, but *mcr* genes disseminated worldwide with an extremely transmissible plasmid (14). However, epidemiological and molecular studies have observed that *mcr*-1 in diversified *Enterobacteriaceae* family includes *K. pneumoniae* (14, 205), *E. aerogenes* (154), *Shigella sonnei* (206), *Enterobacter cloacae* (154)*, Salmonella* (207, 208), *Kluyvera* species (209), *Cronobacter sakazakii* (210), *Citrobacter* species (159, 211), and *Raoultella ornithinolytica* (75) (Table 1). Additionally, *mcr-*1 harboring bacterial isolates exhibited complex resources including human linked environments and natural ecosystem (134, 212–214), food (26, 75, 148, 215), animals (216–218), and human (219–221). The LPS is modified by *mcr*-1 expression by adding cation pEtN transferase (pEtN transferase) (10). However, new variants of *mcr-*1 (*mcr-*1.0 to *mcr-*1.30) were reported with expression by modification of LPS membrane (Figure 4). Additional *mcr* variants were also reported such as *mcr*-2 (*mcr*-2.1 to *mcr-*2.7*)* (135). Phylogenetic studies observed that it is a new variant of *mcr*-1 with 80% identity. Subsequently, three more plasmid-mediated *mcr*-like gene variants were reported in *E. coli* and *Salmonella* as *mcr*-3 (*mcr-*3.1 to *mcr-*3.41) (136), *mcr*-4 (*mcr-*4.1 to *mcr*-4.6) (222), and *mcr*-5 (*mcr*-5.1 to *mcr-*5.4) (149). Phylogenetic studies reported *mcr*-3, *mcr*-4, and *mcr-*5 were descent genes of *mcr*-1/*mcr*−2. In 2018, new *mcr* gene variants, *mcr*-6 (*mcr-*6.1), *mcr*-7 (*mcr-*7.1), and *mcr-*8 (*mcr-*8.1-*mcr*-8.5), were identified that caused increasing spectrum of colistin resistance (143, 144, 223). Carrol et al. reported a novel *mcr* homolog, i.e., *mcr-*9 (*mcr*-9.1 to *mcr*-9.3), in multidrug-resistant colistin-susceptible *S. enterica* serovar *Typhimurium* isolates (150). Surprisingly, *S. enterica* serovar *Typhimurium* strain was phenotypically sensitive to colistin with an MIC value of 2 mg/L, according to (EUCAST) guidelines. Comparison analysis revealed that protein structures of all nine *mcr* homologs (*mcr-*1 to *mcr-*9) depicted that *mcr-*3, *mcr-*4, *mcr-*7, and *mcr*-9 genes have a high degree of resemblance at the structural level (150). Recently, *mcr*-10 (*mcr*-10.1) variant has been identified on an IncFIA plasmid of an *Enterobacter roggenkampii* clinical strain. This *mcr* variant has the highest nucleotide identity (79.69%) with *mcr*-9 and encodes *mcr*-10 with 82.93% amino acids identical to *mcr*-9 (29). The steep increase in plasmid-mediated *mcr* gene variants has raised a serious public health concern during the last few years.

## Current Development to Revert Colistin Resistance

Several approaches are being developed for the treatment of colistin-resistant superbugs (224). To date, three primary approaches to reducing *mcr*-1 associated colistin resistance have been investigated. The first solution is the development of new antibiotics against *mcr* positive organisms, such as eravacycline (225), plazomicin (226), and artilysin (226, 227). Another technique tends to be the standard strategy that involves effective colistin administration and the possible use of combination therapies with additional agents to produce synergistic associations. These agents can include antibiotics that are typically restricted for use against Gram-positive bacteria, such as amikacin (228, 229), aztreonam (229), rifampin (230), azithromycin (230, 231), clarithromycin (232), linezolid (230), azidothymidine (233), tigecycline (234), and derivatives of tryptamine (235). Natural products can also be used to act as adjuvants, some of which might interact with LPSs, such as pentamidine and meridianine D analogs, to disturb the outer bacterial membrane (236, 237). In contrast, other adjuvants do not have specific roles so far, such as resveratrol (238), pterostilbene (239), osthole (240), and niclosamide (241). The last but most important and focused direction is to identify specific drugs targeting *mcr*. There are several methods identified to reduce *mcr* expression at the gene level, such as the use of peptide conjugated phosphorodiamidate morpholino oligomers to target *mcr-*1 mRNA (224), peptide nucleic acid against the *mcr*-1 gene (242), and the CRISPR/Cas9 system to target *mcr*-1 harboring plasmids (243). However, few studies have examined specific drugs targeting *mcr*, with promising results having been observed only for 1-phenyl-2-(phenylamino) ethanone derivatives (244) and the lipid A analog ethanolamine (245). The inhibitory action of ethanolamine against bacteria that produce *mcr*-1 was also tested *in vivo* to further confirm that it can be used as an inhibitor of *mcr*-1 activity. In the presence of 4 mg/mL polymyxin B, the results clearly showed that ethanolamine could inhibit *mcr*-1 expression in a concentration-dependent manner. Furthermore, ethanolamine can be used as an inhibitor of *mcr*-1 activity in light of the structural model and functional unification within the *mcr* family. Reports depicted that ethanolamine acts as an inhibitor of other *mcr* members. However, this would require further experimentation to validate. EptA catalyzes the transfer of PEA from phosphatidylethanolamine to lipid A at 1 and/or 4′ head group positions. EptA is an integral membrane protein consisting of an N-terminal transmembrane domain and a C-terminal soluble periplasmic-facing domain (246, 247). Moreover, EptA enzymes are found in many Gram-negative pathogens, e.g., *E. coli, S. enterica, K. pneumoniae*, etc. (248). In-depth studies are being undertaken to identify and optimize potential EptA inhibitors that suppress expression (224). In addition, similar approaches to EptA inhibition in *Neisseria* species are helpful for developing new therapies. Promising therapies are currently under development to boost phagocytic cells bactericidal activities (249), which could be used as novel combination therapies combined with anti-EptA compounds to effectively decrease transmission of multidrug-resistant bacteria. One strategy to counter this problem is to develop novel antivirulence agents that inhibit lipid A modification by EptA. Inhibition of EptA will hopefully restore the efficacy of polymyxin, support the clearance of infection by the immune system, and minimize the proliferation of colistin resistance (250).

## Future Prospects of Resistance

The emergence of colistin resistance occurs via various mechanisms against the last line defense among carbapenem-resistant Gram-negative bacteria. Several studies led detection of different colistin resistance mechanisms in *Enterobacteriaceae*. However, there is still a lack of knowledge regarding colistin binding and initiating bactericidal activity. As new resistant variants of plasmid-mediated genes emerge that express new mechanisms of LPS modifications, on another side, many bacterial strains have inbuilt intrinsic colistin antibiotic resistance. Such a type of drug resistance occurs by modification of LPS with LAra4N. Moreover, naturally occurring resistant bacteria against colistin were found to have an expression of some specific chromosomal mediated genes such as eptA of *N. meningitides*. It becomes a need of the hour to decipher other possible mechanisms of colistin resistance that are still unknown. Reports revealed that colistin resistance follows only when bacteria are exposed to colistin antibiotics, but other reports suggest that colistin resistance can occur without earlier colistin antibiotic exposure. This represents a serious threat that obstructs the application of colistin as the last line therapy against multidrug-resistant Gram-negative bacteria. A perspective of this phenomenon is crucial and fundamental to protect against the future possibility of the deadly development of bacteria conferring colistin resistance.

## Conclusion

The current epidemiological situation with multiple drug resistance results in the reuse of last-resort antibiotic (colistin) to treat bacterial infection. Moreover, colistin in combination with other drugs becomes a therapy against pathogenic bacteria. The unprecedented use of colistin drug in human medicine, animal husbandry, aquaculture, and agriculture has a serious impact on the emergence and dissemination of colistin resistance among Gram-negative bacteria. The resistance to this lifesaving drug has become a serious public health problem. The primary mechanism of colistin resistance occurs by modulation of chromosomal two-component PmrAB and PhoPQ systems, resulting in modification of the bacterial OM. Additionally, rising risks occur in heteroresistance by colistin attributed in suboptimal colistin dosages and represent a mean potential source of colistin resistance. The emergence of the plasmid-mediated *mcr*-1 gene encoding colistin resistance in bacteria can transfer horizontally from one bacteria to another and further disseminate among animals, humans, and the environment. Moreover, identification of new *mcr* variants such as *mcr*-2, *mcr*-3, *mcr*-4, mcr-5, *mcr-*6, *mcr*-7, *mcr*-8, *mcr-*9, and recently detected *mcr*-10 becomes a more concern to health. There is a clear indication of the rapid spread of plasmid-mediated colistin resistance variants, which requires further studies to evaluate the factors involved, the mechanism of acquisition and dissemination among bacteria. The handier, versatile, and robotic methods are needed to identify different new deadly *mcr* determinants rapidly. Indeed, more investigations are required to identify the exact role of different colistin resistance determinants that will potentially aid in developing new less toxic and potent drugs to treat infections caused by resistant Gram-negative bacteria. Therefore, colistin resistance should be distinguished as a serious medical issue that requires multisectoral research with proper surveillance and suitable monitoring systems to report the rate of dissemination of these resistant genes.

## Author Contributions

FG and QH conceived the idea. FG and MS searched and reviewed the literature and summarized. FG wrote the draft of the paper. FG, MS, and IS contributed to graphics and references. All authors contributed to the article and approved the submitted version.

## Conflict of Interest

The authors declare that the research was conducted in the absence of any commercial or financial relationships that could be construed as a potential conflict of interest.

## Publisher's Note

All claims expressed in this article are solely those of the authors and do not necessarily represent those of their affiliated organizations, or those of the publisher, the editors and the reviewers. Any product that may be evaluated in this article, or claim that may be made by its manufacturer, is not guaranteed or endorsed by the publisher.
